# Potential for plant growth promotion by a consortium of stress-tolerant 2,4-dinitrotoluene-degrading bacteria: isolation and characterization of a military soil

**DOI:** 10.1111/1751-7915.12111

**Published:** 2014-01-28

**Authors:** Sofie Thijs, Nele Weyens, Wouter Sillen, Panagiotis Gkorezis, Robert Carleer, Jaco Vangronsveld

**Affiliations:** Centre for Environmental Sciences, Hasselt UniversityAgoralaan, B-3590, Diepenbeek, Belgium

## Abstract

The presence of explosives in soils and the interaction with drought stress and nutrient limitation are among the environmental factors that severely affect plant growth on military soils. In this study, we seek to isolate and identify the cultivable bacteria of a 2,4-dinitrotoluene (DNT) contaminated soil (DS) and an adjacent grassland soil (GS) of a military training area aiming to isolate new plant growth-promoting (PGP) and 2,4-DNT-degrading strains. Metabolic profiling revealed disturbances in Ecocarbon use in the bare DS; isolation of cultivable strains revealed a lower colony-forming-unit count and a less diverse community associated with DS in comparison with GS. New 2,4-DNT-tolerant strains were identified by selective enrichments, which were further characterized by auxanography for 2,4-DNT use, resistance to drought stress, cold, nutrient starvation and PGP features. By selecting multiple beneficial PGP and abiotic stress-resistant strains, efficient 2,4-DNT-degrading consortia were composed. After inoculation, consortium UHasselt Sofie 3 with seven members belonging to *Burkholderia*, *Variovorax*, *Bacillus*, *Pseudomonas* and *Ralstonia* species was capable to successfully enhance root length of *Arabidopsis* under 2,4-DNT stress. After 9 days, doubling of main root length was observed*.* Our results indicate that beneficial bacteria inhabiting a disturbed environment have the potential to improve plant growth and alleviate 2,4-DNT stress.

## Introduction

The beneficial effects of bacteria on plant fitness have been demonstrated in many environmental conditions including harsh habitats, where the ability of the bacteria to assist plants to overcome these stresses and promote growth has been reported (Weyens *et al*., 2009).

Among abiotic stresses, explosives in soils are a major environmental problem as organic pollutants (Burrows *et al*., 1989; Clausen *et al*., 2004) affecting plant growth and impacting the soil microbial community (Gong *et al*., 1999; Siciliano *et al*., 2000; Travis *et al*., 2008b). Nitro-aromatic explosive contamination is typically found at ammunition production facilities and military sites where co-occurring abiotic stresses such as nutrient limitations, drought stress and cold/hot stress strongly affect the soil restoration capability. 2,4-Dinitrotoluene (2,4-DNT) was produced on a large scale during the World Wars, and it served as a gelatinizing, waterproofing and plasticizing agent in various propellant formulations (Boopathy, 2000; Clausen *et al*., 2004; Riefler and Medina, 2006). The nitro-aromatic is extremely recalcitrant to biological degradation and thus persists in the environment for a very long time (Craig *et al*., 1995; Boopathy, 2000; Brannon and Pennington, 2002). Moreover, 2,4-DNT is toxic and mutagenic to various organisms (Rosenblatt, 1980; Bloch *et al*., 1988; Honeycutt *et al*., 1996; Kilian *et al*., 2001).

One of the major challenges of explosive contaminated sites is their high phytotoxicity and the extreme difficulty of phytotransformation by plants because of the plants' limited catabolic potential to degrade organic contaminants and high phytotoxicity (Makris *et al*., 2010). Studies in recent years highlighted the importance of plant growth-promoting (PGP) bacteria as key players in enhancing the bioremediation/phytoremediation of difficult soils, but only a few reports described the role of PGP bacteria associated with explosives-contaminated military soils. PGP bacteria stimulate the growth of plants in various direct and indirect ways including nutrient provision to plants (Selosse *et al*., 2004; Ryu *et al*., 2005; Hardoim *et al*., 2008; Weyens *et al*., 2009), affecting hormone levels (Arshad *et al*., 2007) and suppressing growth of pathogens (Whipps, 2001).

The investigation of a 2,4-DNT contaminated military soil microbiome might lead to several outputs: (i) understanding the impact of a contaminant on soil micro-organisms, (ii) identification of new 2,4-DNT-degrading strains, (iii) understanding the PGP and stress tolerance of indigenous soil bacteria and (iv) identification of suitable PGP stress-tolerant 2,4-DNT-degrading bacteria for potential use in biological soil clean-up for military lands. To achieve the best results in terms of PGP under stress conditions, it is important to focus on the fraction of cultivable bacteria that is able to thrive already under the specific conditions.

Therefore the aims of this work were (i) to isolate and identify the indigenous soil bacteria of a 2,4-DNT contaminated soil and an adjacent restored grassland soil (GS) of a military training range in Belgium, (ii) to investigate microbial 2,4-DNT degradation, abiotic stress resistance and PGP abilities and (iii) the construction of a new 2,4-DNT-degrading PGP and stress-resistant consortium with potential to sustain plant growth under 2,4-DNT stress.

## Material and methods

### Soil sampling and location

Soil samples were collected from a 2,4-DNT contaminated soil and from an adjacent restored GS at a military training area, located in the North-East of Belgium (Supporting Information Fig. S1). The studied sites were named, DS and GS corresponding respectively to the 2,4-DNT contaminated soil and grassland soil. The 2,4-DNT contaminated soil was a bare soil. Visual inspection of the grass site identified *Molinia caerulea* and *Agrostis capillaris* as the dominant grasses.

Sampling was performed in August and November of 2009 considering different seasonal variations in soil nutrient status and physicochemical properties. Summer and winter samples will be respectively indicated in the text with the codes s and w. Per sampling site, five subsamples (200 g of soil per subsample) were collected at a depth of 0–20 cm with a soil bore. The sieved soil subsamples were mixed in the laboratory to form a composite soil pool with the cultivable bacteria from each site. Analysis of soil nitro-aromatic concentrations were performed as described in EPA method 8330 (1998).

### Biolog ecoplate

One gram of each soil sample was added to 10 ml of phosphate buffer (10 mM, pH 6.8), diluted to 10^−2^ and aliquots of 150 μl were inoculated into each well of the Biolog Ecoplate (Biolog Inc., Hayward, CA, USA). The plates were incubated at 30°C and periodically read with a microplate reader (Biolog Inc.). For a qualitative analysis, the water-well corrected absorbance data were converted into Boolean ones, considering positive (1) the substrates for which absorbance data > 0.10 and negative (0) the substrates for which absorbance data < 0.10. A quantitative analysis was performed by plotting the net area under the curve for each of the 31 response wells over 78 h incubation, according to the trapezoidal approximation (Guckert *et al*., 1996).

### Isolation of 2,4-DNT-tolerant strains

Two different approaches were utilized to obtain 2,4-DNT-tolerant bacteria from the DS and GS military soil. The first approach was performed by a direct isolation technique, starting from a soil suspension (1 g in 10 ml of MgSO_4_) and serial dilutions were spread on 1/10 rich (869) agar plates (Mergeay *et al*., 1985). After 1-week incubation at 30°C, the colony-forming units (cfu) per gram were determined. For the second approach, an enrichment technique was performed whereby 1 g of soil samples was inoculated into 100 ml of selective medium. The selective medium consisted of phosphate buffer (pH 6.8), trace elements, carbon mix (fructose, 1.5; glucose, 1.3; acetic acid, 1 and succinic acid, 0.8 g l^−1^) and 0.1 g l^−1^ 2,4-DNT provided as sole nitrogen source (Snellinx *et al*., 2003). Solid media were prepared by adding 10 g l^−1^ Difco noble agar to the medium. Enrichment cultures were incubated for 3 weeks with shaking at 30°C and diluted with fresh medium when OD_660_ values reached higher than 0.6. At the end of the incubation period, samples were spread on selective medium plates in a way that individual colonies could be isolated. For each sample, between 24 and 50 morphologically different colonies were randomly selected from the plates, purified and used for deoxyribonucleic acid (DNA) extractions.

### DNA extraction, 16S amplified ribosomal DNA restriction analysis (ARDRA) and 16S ribosomal ribonucleic acid (rRNA) gene sequence identification

DNA extraction was performed on each isolated strain using the Qiagen kit (Qiagen N.V., Hilden, Germany). The bacterial collection has been dereplicated through the application of 16S ARDRA polymerase chain reaction using the bacteria-specific primer 26F (AGAGTTTGATCCTGGCTCAG) and the general prokaryotic primer 1392R (ACGGGCGGTGTGTRC) (Barac *et al*., 2004). The 16S digestion products were separated in a gel electrophoresis (90 V, 2 h) with a 1.5% gel, gelred nucleic acid gel stained and visualized under ultraviolet illumination. The 16S ARDRA fingerprint profiles were analyzed to cluster together the bacterial isolates showing the same band pattern. From each cluster, at least one representative strain was selected for auxanography and genotypic identification through 16S rRNA gene sequencing.

Partial 16S rRNA sequences were obtained from Macrogen Inc., Amsterdam, the Netherlands. Nucleotide sequences were subjected to basic local alignment search tool search against the Ribosomal Database. Sequences were aligned with the clustalW function, and an Unweighted Pair Group Method with Arithmetic Mean (UPGMA) phylogenetic tree was constructed with 1000 bootstraps, using the Geneious package (Biomatters Ltd., Auckland, New Zealand). One hundred three partial 16S rRNA gene sequences were submitted to the European Molecular Biology Laboratory (EMBL) database with the accession numbers HG794246-HG794349 (European Nucleotide Archive).

### Use of 2,4-DNT by auxanography

The ability of the isolates to use 2,4-DNT as sole carbon and nitrogen source and as sole nitrogen source was assessed by auxanography on 68 bacterial strains belonging to the different ARDRA groups identified by dereplication. Auxanography is originally described to test the effect of various nutrients on bacteria growth. Here we adapted the method of Parke and Ornston for 2,4-DNT (Parke and Ornston, 1984). Briefly, a washed cell suspension diluted to 10^7^ cfu ml^−1^ was spread on top of solid Noble agar (BD Difco, BD Diagnostic Systems, Sparks, MD, USA) with a minimal medium containing (i) no carbon and no nitrogen to test the use of 2,4-DNT as sole energy source and (ii) with an additional carbon mix to test the use of 2,4-DNT as sole nitrogen source. The plates were dried overnight and then a drop of 2,4-DNT dissolved in dimethylsulfoxide (DMSO) (30 μmol) was spotted onto the plate and spread out in half a circle. Plates with DMSO without 2,4-DNT were used as negative control. Plates were incubated for 1 week at room temperature, and all bacteria were tested in triplicate.

### 2,4-DNT enzyme activity

A microplate assay was used to detect DNT dioxygenase and 4-methyl-5-nitrocatechol (4M5NC) monooxygenase activity (Suen and Spain, 1993). 2,4,6-Trihydroxytoluene (THT) oxygenase activity was detected by suspending cells in 5 ml of phosphate buffer (pH 6.8) with 100 μM of 4-methylcatechol (4MC), which is a structural analogue for THT. The conversion of the medium from colourless to yellow was an indication of THT oxygenase activity. *Burkholderia sp.* JS872 was used as a positive control in the enzyme assays (Nishino *et al*., 2000a). Nitrite release was measured with the Griess assay (Griess Reagent System, Promega, Leiden, the Netherlands).

### Abiotic stress resistance and PGP tests

The ability of the isolated bacteria to cope with drought stress, cold–hot stress and N- and C-starvation was tested on the 31 strains belonging to the 2,4-DNT-tolerant group identified by auxanography (see Supplementary Methods). The potential functionality of these isolates to sustain plant growth was assessed *in vitro* by a large screening for PGP abilities using specific media (see Supplementary Methods).

### Combinations of 2,4-DNT-degrading consortium members

2,4-DNT-degrading consortia were constituted by adding 0.5 ml of twice MgSO_4_-washed suspensions of overnight grown bacterial cells on rich medium, into 100 ml of selective medium containing 2,4-DNT (500 μM) as sole nitrogen source. Many different combinations of abiotic stress resistant and PGP-promoting strains were made.

The disappearance of 2,4-DNT in liquid cultures was determined using HPLC [Agilent 1100; C18 reverse-phase column (5 μm, 250 × 4.6 mm)]. To 500 μl of culture supernatant, 500 μl of methanol was added, mixed and filtered through a 0.45 μm polytetrafluoroethylene filter before analysis. Control samples were taken from sterile 2,4-DNT medium.

### Root growth promotion under 2,4-DNT stress on vertical agar plates system (VAPS)

The bacterial consortia were inoculated onto VAPS with *Arabidopsis thaliana* seedlings to study root growth responses. In the Supplementary methods a detailed description of the plant cultivation and inoculation is given. The plant experiments were performed with 15 biological replicates. The statistical analysis was performed by analyzing the data with analysis of variance (*P* < 0.01) using the r programme and Tukey's honest significant difference for multiple comparisons. Non-normality and unequal variance data were analyzed with Kruskall–Wallis.

## Results and discussion

### Soil conditioning and nitro-aromatic concentrations

The 2,4-DNT contaminated soil (DS) and the GS, were both classified as a sandy soil. For an overview of the physicochemical soil parameters (pH, conductivity, organic carbon content) see Supporting Information Table S1. High-performance liquid chromatograph (HPLC) analysis revealed high concentrations of 2,4-DNT in the contaminated soil (74.8 and 83.4 mg kg^−1^ respectively in summer and winter) whereas low concentrations were detected in the adjacent GS (< 1.52 mg kg^−1^) (Supporting Information Table S1). Related amino-derivates and co-contaminants were present in the DS but not in the GS. 2,4,6-TNT was not detected. The contaminated soil was mainly used for disposal of hazardous dinitrotoluene-manufacturing wastes and burning ammunition including fireworks. Because the Belgian army did not use the nitramines 1,3,5-Trinitro-1,3,5-triazacyclohexane (RDX) and Octahydro-1,3,5,7-tetranitro-1,3,5,7-tetrazocine (HMX) in explosives formulations, we did not analyze the soils for these compounds.

Summer samples were collected in August of 2009 characterized by low total amount of rain (63.8 mm in comparison with a normal value of 144.2 mm). Winter samples were collected in November of 2009 characterized by normal values for average temperature (3.5°C), amount of rain (163.3 mm) and number of rain days (45).

### Ecocarbon substrate use analysis of the microbial communities

Biolog Ecoplates (Biolog Inc.) provide a rapid screening of soil communities in terms of Ecocarbon use. Here, the Biolog Ecoplates were used to give a snapshot of the metabolic activity of the cultivable bacterial assemblages in the 2,4-DNT contaminated bulk soil and from an adjacent restored grass site soil. Summer and winter inoculums for the grass site were characterized by a diverse and metabolic active bacterial community, metabolizing 26 and 27 out of 31 Ecocarbon substrates respectively (Supporting Information Table S2). The 2,4-DNT contaminated summer and winter communities were positive for 24 and 19 carbon sources respectively being negative for seven substrates namely 6, 7, 8, 9, 14, 18 and 22. The more Ecocarbon sources that can be used, the more diverse the community. As such, the results indicate that the contaminated soil has a less diverse bacterial population than the adjacent grass site. A scatterplot of the Biolog Ecoplate responses (Supporting Information Fig. S2) indicated that the microbial community from the contaminated soil has a reduced ability to utilize carbon sources 11, 16, 17, 24, 25, 27 and 31 in comparison with the grass site. Taken together, the Biolog Ecoplates (Biolog Inc.) suggest a less diverse and less metabolic active bacterial community in the 2,4-DNT contaminated soil than the restored grass site. It has been shown before that environmentally disturbed soils have a reduced Ecocarbon substrate use (Travis *et al*., 2008a).

### Isolation and characterization of the cultivable bacteria

**Fig. 1:**
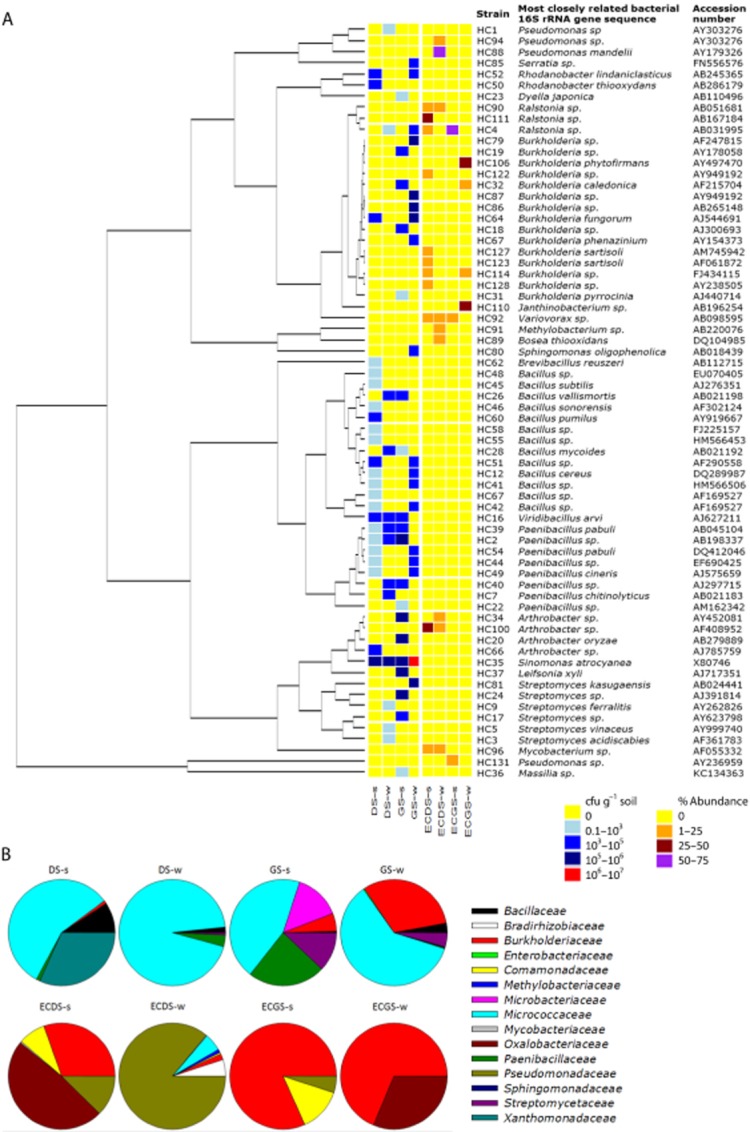
A. UPGMA tree and heat map showing the phylogenetic relationships and abundance of the isolated strains for each of the different soils. The identified species correspond to the 68 clusters of different 16S rRNA gene ARDRA profiles, grouped based on 99% nucleotide similarity of the 16S rRNA gene sequences. From left to right: the UPGMA phylogenetic tree constructed with Geneious using the 16S rRNA gene sequences, heat map showing the abundance of the species in cfu g^−1^ soil for DS and GS obtained on 1/10 rich medium (yellow: 0 cfu g^−1^; light blue: 0.1–10^3^ cfu g^-1^, blue: 10^3^–10^5^ cfu g^−1^, dark blue: 10^5^–10^6^ cfu g^−1^ and red: 10^6^–10^7^ cfu g^−1^) and the relative abundances in % of the species in each soil after enrichment cultures (EC) of the soils in the colour codes (yellow: 0; orange: 1–25%; dark-red (25–75%) and purple (75–100%). The corresponding strain number is shown next to the heat map, the most closely related bacterial 16S rRNA gene sequence and Genbank accession number of the most closely related species. B. Pie diagrams showing the bacteria family distribution of the different soils (in percentage %) on 1/10 rich medium (DS-s,w; GS-s,w) and isolated after successive dilution cultures with 2,4-DNT [enrichment culture 2,4-DNT contaminated soil (ECDS)-s,w; ECGS-s,w]. Calculations were performed on the different 16S rRNA gene identified strains.

In a first approach, the cultivable bacteria were isolated on heterotrophic 1/10 rich medium from the DS and GS collected soil samples at the military site. The abundance of cultivable bacteria (Fig. 1 and Table 1) was considerably variable between the two sites. The number of cfu per gram soil was 10 times lower for the bare 2,4-DNT contaminated site [2,4-DNT contaminated soil summer (DS-s): 2.43 ± 0.08 × 10^5^; 2,4-DNT contaminated soil winter (DS-w): 2.3 ± 0.12 × 10^5^ in comparison with the bulk soil samples from the adjacent grass site [grassland soil summer (GS-s): 3.25 ± 0.025 × 10^6^; grassland soil winter (GS-w): 1.06 ± 0.1 × 10^6^] indicating 2,4-DNT toxicity for bacteria. A seasonal effect on cfu count was only observed for the GS, showing three times higher cfu count in summer than winter. We collected bulk soil samples in the grass site to exclude the specific rhizosphere effect and to allow comparison with the non-vegetated contaminated soil. However, the presence of plants releasing photosynthetic exudates in soil is known to sustain a larger microbial soil population (Van Dillewijn, 2008). Nevertheless, this work genotypically and phenotypically characterized the micro-organisms in a 2,4-DNT contaminated field soil and a neighbouring restored grass site, with the purpose of isolating and identifying new plant growth-promoting, stress-resistant and 2,4-DNT-degrading bacteria, which can be applied as a biofertilizer to increase plant growth on military ranges. We established a collection of 146 cultivable isolates associated with DS and GS. 16S ARDRA fingerprinting was applied to dereplicate the bacterial collection allowing the identification of 68 clusters corresponding to different ARDRA profiles and representing different species (Fig. 1). From each cluster, at least one representative strain was sent for sequencing. In total, 103 partial 16S rRNA gene sequences (1000 bp) were obtained and submitted to the EMBL database with the accession numbers HG794246-HG794349 (European Nucleotide Archive).

**Table 1:** Diversity indexes of the cultivable bacteria collection isolated on 1/10 rich medium of the 2,4-DNT contaminated soil and grassland soil in summer and winter

Parameter	2,4-DNT contaminated soil		Adjacent grassland soil	
	
	DS–s	DS-w	GS–s	GS–w
Genera	8	7	10	9
Individuals(cfu g^−1^soil)	2.43 ± 0.08 × 10^5^	2.3 ± 0.12 × 10^5^	3.25 ± 0.05 × 10^6^	1.06 ± 0.1 × 10^6^
Shannon–Wiener	1.65	1.02	2.5	1.46
Evenness	0.58	0.38	0.82	0.55
Species richness	23	13	19	18

Shannon–Wiener and evenness indexes are calculated for the 16S rRNA gene sequences of the individual species in the different analyzed soils.

The taxonomic identification of the bacteria in the contaminated soil in summer showed a high dominance of the Micrococcaceae, Xanthomonadaceae and Bacilli (Fig. 1 and Table 1). Among the Micrococcaceae, the most abundant species retrieved was *Sinomonas atrocyanea*. For DS-s, the highest species richness (23) was recorded from which the genera *Paenibacilli*, *Bacilli* and *Burkholderia sp.* were numerously represented. Nevertheless, the Shannon diversity index (1.02) and evenness (0.38) were low. Grassland soil-s harboured the highest biodiversity on the genus level (10) and species were more evenly distributed reflected by a high Shannon diversity index (2.5) and evenness (0.82) (Table 1). In the winter bacteria collection, the contaminated soil and the grass site were strongly dominated by the Micrococcaceae, which resulted in a lower evenness (0.5).

For the isolation of 2,4-DNT-tolerant strains, a selective enrichment approach was applied and 16 species were obtained from the 2,4-DNT contaminated soil whereas only seven isolates were obtained from the grass site. Figure 1 reveals a more diverse 2,4-DNT-tolerant subpopulation on the family level obtained from DS than GS for both summer and winter after successive culturing on 2,4-DNT. The most dominant 2,4-DNT-tolerant genera in the contaminated soil are *Pseudomonas*, *Burkholderia*, *Variovorax* and *Janthinobacterium*.

The sandy military soils studied here, hosted a diverse bacterial community comprising different genera that were previously reported in other soil environments (Janssen, 2006). The Micrococcaceae including *Arthrobacter* and *Sinomonas*, Bacillaceae, Paenibacillaceae and *Pseudomonadaceae* are often abundant genera in soils isolated by cultivation techniques (Janssen, 2006). The genera *Burkholderia, Pseudomonas*, *Ralstonia* and *Variovorax* represent an important taxonomic group typical of nitro-aromatic contaminated soils (Nishino *et al*., 2000b; Snellinx *et al*., 2003). The taxonomic analyses of the bacteria isolated from the bare 2,4-DNT contaminated soil and adjacent grassland of the same military site permitted to assess the effect of intra-site specific disturbances such as 2,4-DNT on the composition of the cultivable heterotrophic community. Even though isolation on 1/10 rich medium resulted in a higher cfu count and richer taxonomic diversity of GS-isolates, a higher degree of 2,4-DNT-tolerant bacteria was isolated after enrichments on selective medium from the DS-site. It has been shown before that 2,4-DNT and TNT contamination in soils can influence the prevalence of metabolizing and tolerant strains (Snellinx *et al*., 2003; Travis *et al*., 2008a,b)

### Use of 2,4-DNT by auxanography

**Fig. 2:**
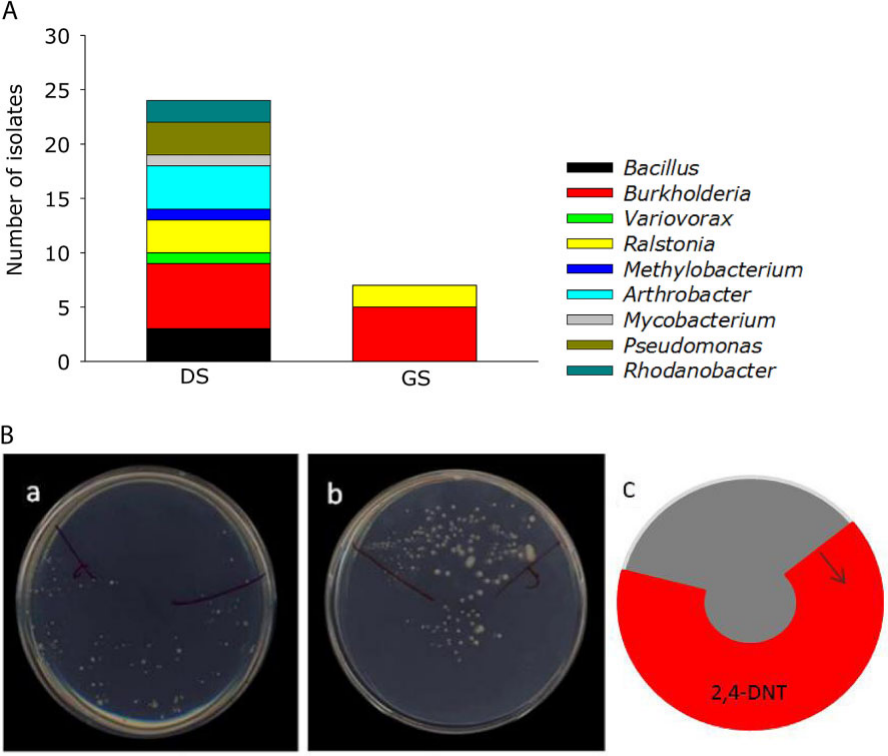
A. Number of isolates grouped per genus, scoring positive for auxanography using 2,4-DNT as sole N-source. DS: 2,4-DNT contaminated soil summer and winter combined, GS: grassland soil summer and winter combined. B. Positive auxanography response towards 2,4-DNT as sole N-source by *Ralstonia sp.* HC90 (a) and negative auxanography response towards 2,4-DNT by *Paenibacillus sp.* HC2 (b). A sample of bacteria in MgSO_4_-buffer (100 μl of 10^7^ cfu ml^−1^) was spread evenly over the whole surface of the minimal agar plate. After drying overnight, a drop of 2,4-DNT (30 μmol dissolved in DMSO) was spot on the plate and spread-out in half a circle, as shown in the scheme (c). After 5 days of incubation at 30°C, growth of bacteria was observed as white colonies. *Ralstonia sp.* HC90 grows only on the 2,4-DNT treated zone of the plate (+); *Paenibacillus sp.* HC2 strictly avoided growth on the 2,4-DNT treated zone (−).

Aiming to identify 2,4-DNT transforming bacteria for 2,4-DNT biodegradation, the ability of the isolated bacteria to grow in the presence of 2,4-DNT as sole nitrogen and energy source was tested on 68 bacterial strains belonging to the different ARDRA groups. The strains able to form colonies on the 2,4-DNT streaked zone were scored positive. None of the individual strains could use 2,4-DNT as the sole energy source on the auxanography plates. However, for 2,4-DNT applied as sole nitrogen source, we identified several positive strains and they were mostly associated with the exposure of bacteria in soil to 2,4-DNT (Fig. 2). In Fig. 2B a positive and negative auxanography response towards 2,4-DNT by bacteria is shown. All tests were performed in triplicate, and no bacterial growth was observed on the negative control plates containing only DMSO.

### 2,4-DNT enzyme assay

Previous studies indicate that all 2,4-DNT-degrading bacteria use the same oxidative steps to mineralize 2,4-DNT, involving an initial 2,4-DNT dioxygenase resulting in the formation of 4M5NC and the release of nitrite. The results from the auxanography test suggest that none of the individual strains can mineralize 2,4-DNT. To provide any evidence of the possible presence of genes encoding 2,4-DNT dioxygenase and enzymes involved in the lower pathway such as 4M5NC monooxygenase and 2,4,5-THT oxygenase activity, we performed an enzyme activity tests on the subcollection of 2,4-DNT-tolerant strains retrieved from the auxanography test. None of the strains exhibited 2,4-DNT dioxygenase activity. 4M5NC activity was demonstrated in three strains, *Burkholderia phytofirmans* HC106, *Pseudomonas sp.* HC88 and *Ralstonia sp.* HC90 as indicated by the detection of nitrite. Furthermore, one strain *Burkholderia sp.* HC114 was able to oxidize 4MC shown by the concomitant colour change of the medium from transparent to yellow. We used 4MC as structural analog of 2,4,5-THT in the enzyme test because dissolving THT in the medium gave already a pink coloration indicating auto-oxidation to hydroxymethylquinone. The bacterial oxidation of 4MC and 2,4,5-THT was confirmed with HPLC. As a positive control, we incorporated *Burkholderia cepacia* JS872 (Nishino *et al*., 2000a).

### Resistance to abiotic stresses and PGP abilities

Because we are interested to identify the most suitable soil bacteria for use as ‘biofertilizer’ to sustain plant growth on the disturbed military soil, the ability of the isolated bacteria to cope with drought stress, cold–hot stress and N- and C-starvation, typical for sandy military soils, and the potential to promote plant growth was tested on the 31 strains belonging to the 2,4-DNT-tolerant group identified by auxanography.

The strains able to grow at 50°C represented 86.7% of the total strains and the ability to grow at low temperature (4°C) was observed for 64% of the isolates (Table 2). The open landscape, regular bush fires and the poor vegetation on explosives soils are factors that contribute to more extreme temperature fluctuations affecting the soil. In this view, the ability of the military soil isolates to survive under a broad temperature range is useful for efficient colonization of barren habitats.

**Table 2:** Plant growth-promoting potential and abiotic stress resistance of the microbial subcollection.

genus	Nr. of isolates	Strain HC	PGP activities and tolerances to abioticstress (%)					ACCd	4°C	50°C	Average % survival after 14d of water stress	Average % survival after 14d of N- and C-starvation

			IAA	P Sol.	Sid.	Org. Acid	Acetoin					
*Arthrobacter*	3	34;66;100	100	100	0		0	33	100	100	37.6 ± 1.13	
*Bacillus*	2	26;45	50	100	0	100	0	100	100	50	<5.0 ± 1.2	80.6 ± 3.2
*Burkholderia*	11	19;18;31;32;64;7		100	45	100	0	100	75	100	35.5 ± 2.4	74.5 ± 2.2
		9;106;114;122;12 9										
		3;128										
*Methylobacterium*	1	91	100	0	0	100	0	100	0	100	42.2	52.2
*Mycobacterium*	1	96	100	0	0	100	0	100	0	100	47.6	57.6
*Pseud omonas*	4	1;88;94;131	75	50	100	100	0	100	50	50	62.2 ± 2.3	62.2 ± 1.3
*Ralstonia*	3	4;90;111	0	0	100	0	100	100	50	100	49.8 ± 1.2	69.8 ± 2.2
*Rhodanobacter*	2	50;52	100	100	0	0	0	0	100	100	82.5 ± 1.3	92.5 ± 1.2
*Sinomonas*	1	35	0	0	0	0	100	100	100	100	92.4	95.4
*Vario vorax*	1	92	100	100	100	100	0	100	100	100	87.5	89.5
*Viridibacillus*	1	16	0	100	0	0	0	100	0	0	81.6	72.7
TOTAL (%)			43.3	73.3	43.2	66.7	13.3	86.6	64.0	86.7		

The PGP activities are expressed in percentage of isolates within each genus that score positive for the test. In the last row, the total percentage of isolates scoring positive for PGP activities and tolerance to abiotic stress is shown. P Sol., inorganic phosphate solubilization; Sid., siderophores production; Org. Acid, organic acid production; AACd, ACC deaminase activity.

A high percentage of the isolated bacteria was able to survive a 2-week period of drought stress (Table 2). Sparse vegetated and completely barren soils on military sites are most prone to drought stress. The summer of 2009 had a low amount of total rain. Soil drying causes adverse physiological effects on soil bacteria associated with cell dehydration. Some genera or bacteria are more tolerant than others. From Table 2, we can observe that Bacilli were the most water tolerant. This is not completely unexpected as *Bacillus sp.* are capable of forming endospores that allow bacterial survival for extended time periods under adverse conditions.

Many sandy soils in the North-East of Belgium, including the studied military soil, suffer from nutrient stress. Therefore the ability of the isolates to survive a period of N- and C-limiting conditions was assessed. The response distribution among the different genera revealed that *Rhodanobacter sp.* flourished best under nutrient-limiting conditions. *Rhodanobacter sp.* HC50 and *Pseudomonas sp.* HC94 showed blue-stained lipid granules in the cytoplasma after 14 days of nutrient starvation (Supporting Information Fig. S3). These lipid granules contain poly-β-hydroxybutyrate and are an important source of food during starvation.

The tolerance to abiotic stresses including temperature fluctuations, drought stress and the ability to thrive under nutrient-limiting conditions was found to be widespread within the military soil bacteria collection and represented a common trait even in phylogenetic unrelated strains.

### Plant growth promotion ability

The potential functionality of the 31 selected soil isolates to sustain plant growth was assessed *in vitro* by a large screening for PGP abilities using specific media.

One of the strategies adopted by PGP bacteria to induce plant growth is the influence on the plant hormonal balance. From the 2,4-DNT-tolerant strains, 43.3% was able to produce indole-3-acetic acid (IAA), an important hormone of the auxin family (Table 2). This trait was most expressed among *Arthrobacter*, *Bacillus*, *methylo-* and *mycobacterium*, *Pseudomonas*, *Rhodanobacter* and *Variovorax* members. The emission of the volatile plant hormone compound acetoin was only shown for the genera *Ralstonia* and *Sinomonas*.

Plant growth-promoting bacteria can also indirectly influence the health status of the plant by reducing the concentration of the stress signalling molecule 1-aminocyclopropane-1-carboxylate (ACC), a precursor of ethylene. Nine out of eleven genera of the collection tested positive for ACC-deaminase activity (ACCd), only members of *Arthrobacter* and *Rhodanobacter* were unable to convert ACC. The high percentage of ACCd among the military soil isolates is in agreement with previous studies reporting the occurrence of ACCd in various soil bacteria (Grichko and Glick, 2001; Glick, 2005; Arshad *et al*., 2007).

Direct mechanisms of plant growth promotion also include those making nutrients more available to plants and in that way improve plant fitness. The bacterial collection was investigated for the capability to solubilize phosphate, produce organic acids and siderophores for making iron more bioavailable. Of the isolates, 73.3% were able to solubilize phosphorous as observed by the clear halo's on plates (Supporting Information Fig. S4). Siderophore production was positive for 43.2% of the isolates including *Burkholderia*, *Pseudomonas*, *Ralstonia* and *Variovorax*. Organic acid production activity was present in 66.7% of the isolates including members of *Bacillus*, *Burkholderia*, *Methylo*- and *Mycobacteria*, *Pseudomonas* and *Variovorax*. The widespread ability to increase the concentration of bioavailable nutrients in the isolate collection suggests the contribution of these strains to the plant nutrient balance.

Besides direct PGP activity, we observed that *Ralstonia sp.* HC90 displayed flagella and demonstrated swimming behaviour in semi-solid plates (Supporting Information Fig. S5A and D). In addition, this strain demonstrated chemotaxis towards 2,4-DNT observed by the yellow chemotactic ring on the plates (Supporting Information Fig. S5B). In soil, chemotaxis is a beneficial feature bringing motile-degrading bacteria into close contact with the substrate, stimulating biodegradation.

The results about the investigation of the PGP potential demonstrate that a majority of the bacteria harboured multiple PGP traits. The PGP traits distribution among the different bacterial genera revealed that *Burkholderia*, *Pseudomonas* and *Variovorax* showed a predominant role, displaying almost all PGP traits investigated. The simultaneous presence of several PGP features in the same strain possibly act in a synergic way to promote plant growth.

### Compiling a new 2,4-DNT-degrading and stress-tolerant consortium with PGP potential

With the aim of constructing a new 2,4-DNT-mineralizing consortium, we incorporated *Variovorax paradoxus* strain VM685 to our study catalyzing the initial oxidation of 2,4-DNT (Snellinx *et al*., 2003). The strain was isolated in 2003 from an ammunition brownfield, located 30 km from the military training range. The soil isolate is phylogenetically closely related to the military soil isolates. *Variovorax paradoxus* contains a gene similar to *dntA*, a 2,4-DNT dioxygenase and *dntB*, 4M5NC monooxygenase activity. The enzyme activity was confirmed in the microplate assay based on the detection of nitrite and by HPLC. No transformation beyond 4M5NC, 2-hydroxy-5-methylquinone (2H5MQ) and 2,4,6-THT was observed in liquid cultures with *V. paradoxus* VM685 under carbon limiting conditions. This is expected because *V. paradoxus* does not harbour these genes of the degradation pathway.

Because the effectiveness of a microbial consortium is highly dependent on its competitiveness in soil and root colonization, the resistance of the military soil isolates to the abiotic stresses, and the expression of beneficial PGP traits is a prerequisite to compile new 2,4-DNT-degrading consortia. Constitutions with all possible combinations of (i) the newly identified 4M5NC and 2,4,6-THT-degrading military soil isolates and (ii) strains selected based on their promising multiple PGP activities and the ability to cope with several abiotic stresses and (iii) *V. paradoxus* VM685 were constructed. The ability of the consortia to metabolize 2,4-DNT (500 μM) was assayed under carbon limiting conditions.

**Fig. 3:**
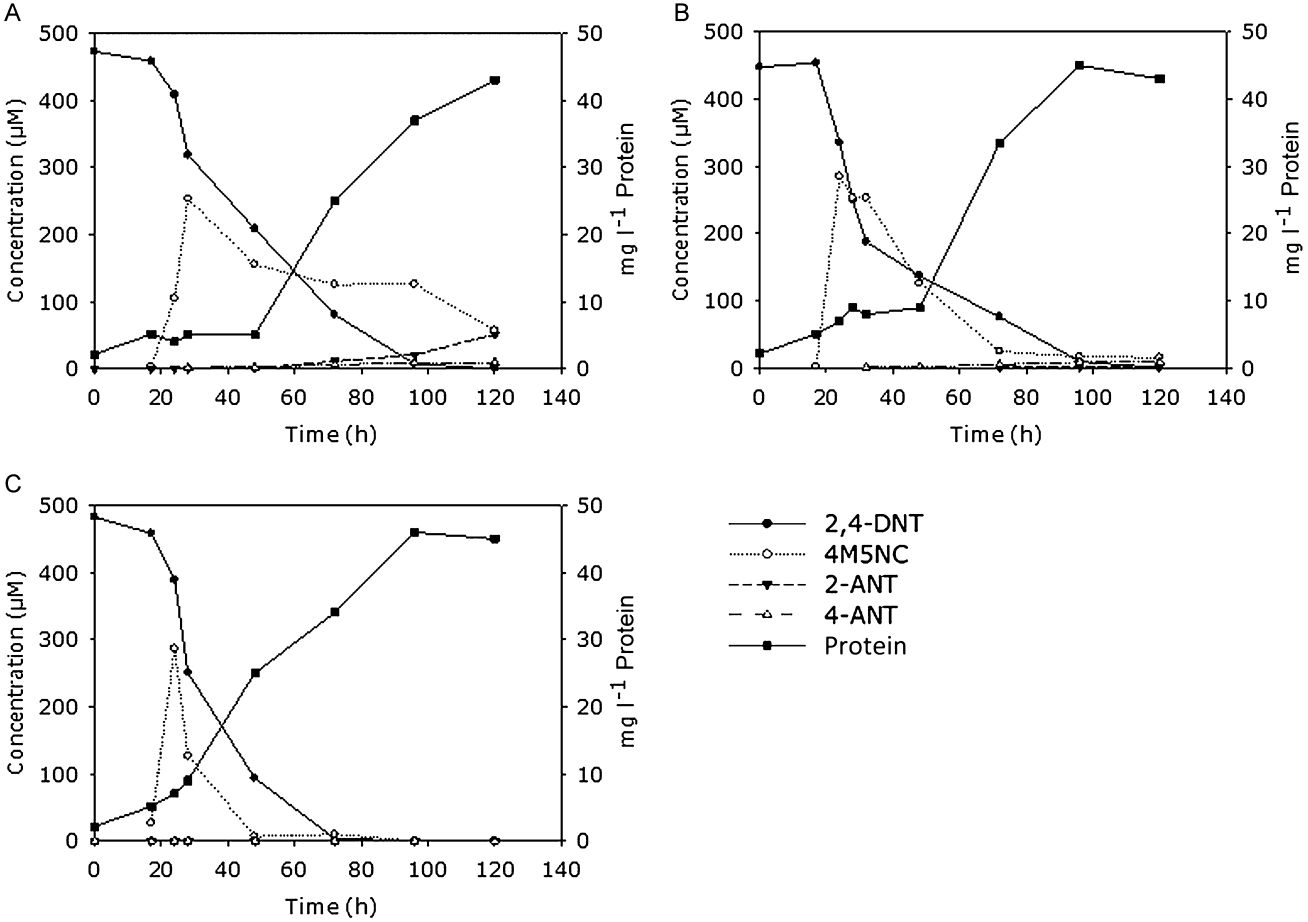
Metabolization of 2,4-DNT by three consortia in liquid cultures under carbon limiting conditions (500 μM of 2,4-DNT was added as sole energy source). Y-axis: (left) concentration of the explosives in μM; (right) bacterial protein concentration (mg l^−1^) measured by the Bradford assay; X-axis: time in hours.A. Consortium UHS1 (*Pseudomonas mandelii* HC88, *V. paradoxus* VM685 and *Burkholderia sp.* HC114).B. Consortium UHS2 (*V. paradoxus* *VM685;* *Burkholderia sp.* HC114, *Pseudomonas mandelii* HC88, *Ralstonia sp.* HC90 and *Burkholderia phytofirmans* HC106).C. Consortium UHS3 (*V. paradoxus* *VM685;* *Burkholderia sp.* HC114, *Pseudomonas mandelii* HC88, *Ralstonia sp.* HC90, *Burkholderia phytofirmans* HC106, *Bacillus subtilis* HC45 and *Variovorax sp.* HC92).

The HPLC results demonstrated that various multiple-member consortia could degrade 2,4-DNT, but the presence of *Burkholderia*. HC114 and *V. paradoxus* VM685 were essential. Cultures without *V. paradoxus* exhibited reduction reactions leading to the formation of 2-A-4NT and 4-A-2NT, but no growth was observed. Cultures with *V. paradoxus* and *Burkholderia sp.* HC114 accumulated 4M5NC and nitrite in the medium and no continuous growth and degradation beyond 4M5NC was observed (data not shown). The best three-member consortium UHS1 was compiled of *V. paradoxus VM685*, *Burkholderia sp.* HC114 in combination with *Pseudomonas mandelii* HC88. 2,4-DNT was completely degraded in 4 days, and no nitrite was detected. However, small amounts of 4M5NC (< 89 μM) still persisted in the medium after 4 days and also small amounts of the reduction products 2-A-NT and 4-A-NT (< 50 μM) were detected with HPLC (Fig. 3A). Consortium UHS2 was compiled of the beneficial members of consortium UHS1 (*V. paradoxus VM685*; *Burkholderia sp.* HC114 and *Pseudomonas mandelii* HC88) in combination with *Ralstonia sp.* HC90 and *Burkholderia phytofirmans* HC106. Degradation was completed in 4 days, continuous growth was observed beyond 4M5NC and none of the reduction products were detected with HPLC (Fig. 3B). However, growth was only increased when 4M5NC started to decline significantly in the medium after 2 days, indicating that high concentrations of 4M5NC inhibit bacterial growth. Consortium UHasselt Sofie 3 (UHS3) was composed of the beneficial members of consortium UHS2 with *Bacillus subtilis* HC45 and *Variovorax sp.* HC92*.* Degradation took 3 days; no major reduction products accumulated and growth increase started earlier in relation to a faster decrease in 4M5NC (Fig. 3C).

### *In vitro* plant growth promotion of military soil consortia under 2,4-DNT stress

**Fig. 4:**
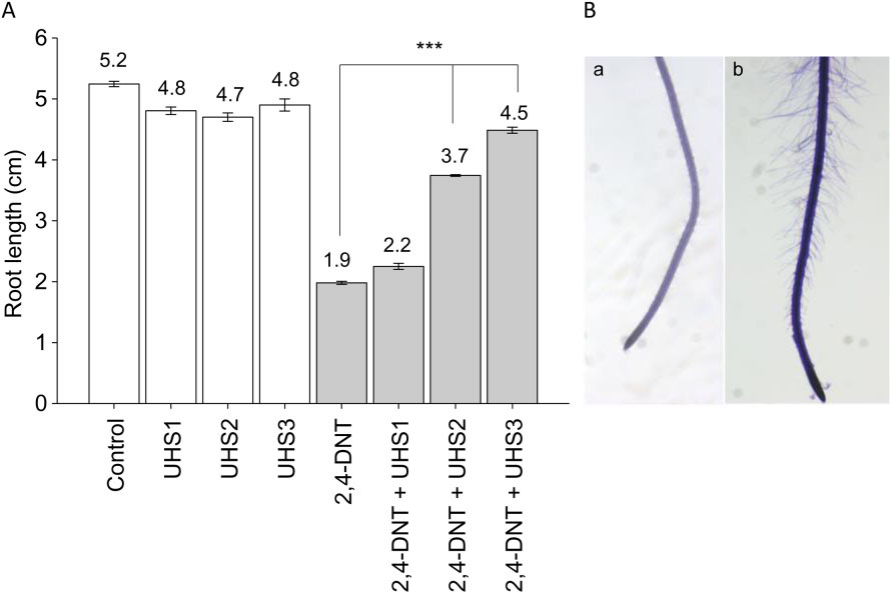
A. Root length of *A. thaliana* seedlings non-exposed (bars in white) and exposed to 1 mg l^−1^ 2,4-DNT for 9 days (bars in grey) in the absence or presence of consortia UHS1, UHS2, UHS3 (10^6^ cfu ml^−1^). Values are mean ± standard error of at least 15 biological replicates. (Significance level versus control: *** *P* < 0.001, non-parametric Kruskal–Wallis test). B. Pictures of root hairs were taken under the binocular (20×) of (a) un-inoculated 2,4-DNT exposed plants and (b) 2,4-DNT exposed plants inoculated with UHS3.

The three consortia, UHS1, UHS2 and UHS3, which were successful for total degradation of 2,4-DNT in liquid cultures, were evaluated for PGP potential under 2,4-DNT stress on VAPS with *A. thaliana* (Fig. 4). The growth medium for the plant was a C-free minimal medium, but the inoculated PGP bacteria were probably not suffering from C-starvation as we assume that they were able to use the plant root exudates as carbon and nitrogen source.

For the inoculation experiments, we have chosen to work with 1 mg l^−1^ 2,4-DNT because this concentration reduced root length by 80% in comparison with the non-exposed plants. The plant results showed that consortia UHS2 and UHS3 were able to remarkably increase primary root length by more than 60% in comparison with the non-inoculated 2,4-DNT-exposed plants. The highest root growth promotion was observed for consortium UHS3, with a significant doubling in main root length (4.5 ± 0.04 cm to 1.9 ± 0.05 cm, respectively, *P* < 0.001) (Fig. 4 and Supporting Information Fig. S6). An important finding was that total root length of the exposed plants inoculated with consortium UHS3 equalled that of the unexposed plants (4.8 ± 0.12 cm), hence this consortium totally relieved the negative effect of 2,4-DNT on plant root elongation. We also noticed that the root hairs of UHS3 inoculated plants were significantly longer and denser in comparison with the control plants (Fig. 4B). At the end of the growth study, the shoot parts of the plants inoculated with consortium UHS3 were surface sterilized, and the endophytic bacteria were isolated. The box-fingerprint patterns of two reisolated strains (RI2 and RI8) matched those of *Ralstonia sp.* HC90 and *Pseudomonas mandelii* HC88, indicating that these soil isolates are facultative endophytes (Supporting Information Fig. S7). A possible mechanism by which the bacteria could enter the plant is during root hair formation. Moreover *Ralstonia* was flagellated, an important trait for endophytic root colonization.

The members of consortium UHS3 were able to grow under different stresses (high and low temperatures, nutrient starvation and drought stress, and to perform PGP activities *in vitro* and also showed to enhance primary root growth *in vivo* under 2,4-DNT stress (Table 3). The added value of working with consortia is that bacteria can stimulate the activity and growth of other strains by metabolic cross-feeding, e.g. by nutrient transfer or more rapid degradation of toxic intermediates (Snellinx *et al*., 2003). Another added value of working with consortia, and this is less commonly investigated, is the possible synergistic action of the beneficial PGP traits of the individual consortia members, eliciting a positive plant growth response. Here, we suggest that a synergistic PGP action is involved in the root-lengthening effect observed under 2,4-DNT stress for *A. thaliana* inoculated with consortium UHS3.

**Table 3:** Composition and characteristics of consortium UHS3. Scores are in (+) and (−) representing the capability respectively incapability to display the trait

Consortium UHS3	Most closely related bacterial 16S rRNA gene sequence	Accession number of the most closely related 16S bacterium	Conversion of the following substrate			PGP activities and tolerances to a biotic stress								% survival after 14 days of drought stress	% survival after 14d of N- and C-starvation
	
			2,4–DNT	4M5NC	4MC	IAA	P sol.	Sid.	Org. Acid	Acetoin	ACCd	4°C	50°C		
HC45	*Bacillus subtilis*	AJ276351	−	−	−	−	+	−	+	−	+	+	+	< 5.0 ± 1.8	80.6 ± 3.2
HC106	*Burkholderia phytofirmans*	AY497470	−	+	−	−	+	+	+	−	+	+	+	35.5 ± 2.4	74.5 ± 3.7
HC114	*Burkholderia sp.*	FJ434115	−	−	+	−	+	−	+	−	+	+	+	89.5 ± 2.2	58.9 ± 2
HC88	*Pseudomonas mandelii*	AY179326	−	+	−	+	+	+	+	−	+	+	+	63.1 ± 1.7	67.6 ± 2.3
HC90	*Ralstonia sp.*	AB051681	−	+	−	−	−	+	−	+	+	+	+	49.8 ± 3.1	69.8 ± 4.1
HC92	*Variovorax sp.*	AB098595	−	−	−	+	+	+	+	−	+	+	+	87.5 ± 1.1	89.5 ± 2.4
VM685	*Variovoraxparadoxus*	–	+	+	−	+	+	−	−	−	−	+	+	47.3 ± 4.5	56.2 ± 1.8

## Conclusions

Ecocarbon source profiling of the 2,4-DNT contaminated soil and the grass border showed that the presence of 2,4-DNT and absence of vegetation on the barren soil were the driving forces that changed the carbon substrate use of the bacterial community. Isolation of the cultivable bacteria from the 2,4-DNT contaminated soil showed a lower cfu count and a less diverse microbial community.

After selective enrichments a larger collection of 2,4-DNT-tolerant strains was isolated from the contaminated soil and their identification and further characterization with auxanography broadened our knowledge on the DNT-tolerant bacteria in the military soil. Furthermore the characterization of a large collection of soil bacteria that (i) tolerate drought stress, cold, hot temperature, nutrient starvation and (ii) showed *in vitro* the ability to positively influence the plant growth are novel findings in this study. Also the endophytic plant colonization by two soil isolates, a flagellated, chemotactic *Ralstonia sp.* HC90 and *Pseudomonas sp.* HC88 was observed. Furthermore, we reconstituted new 2,4-DNT-degrading consortia by selecting bacteria based on the promising multiple PGP activities *in vitro* and the ability to cope with several abiotic stresses. A seven-member consortium UHS3 showed the ability to quickly degrade 2,4-DNT in liquid cultures and remarkably increase primary root length and root hairs of 2,4-DNT exposed plants *in vivo*. This demonstrates the suitability of this military soil consortium to set up effective PGP-inocula *in situ*. The great potential of PGP bacteria and stress-resistant bacteria should be taken into consideration when aiming for sustained 2,4-DNT degradation and plant growth under harsh field conditions. The present work represents a pool of beneficial 2,4-DNT-degrading PGP, stress-tolerant bacteria in consortia able to enhance plant growth in disturbed military soils impacted by 2,4-DNT contamination and abiotic stresses.

## Supporting information

**Fig. S1.** Photographs of the investigated field soils at the military training range in the North-East of Belgium. Left, the bare 2,4-DNT contaminated soil with bomb remnants and detonation container. On the left side, the adjacent grassland soil colonized by *Molinia caerulea* and *Agrostis tenuis*, photograph was taken in November 2009.

**Fig. S2.** Scatterplot of Biolog EcoPlate responses (average net area under the curve for each of the 31 carbon sources) comparing the grassland soil winter (GS-w), 2,4-DNT contaminated soil summer (DS-s) and winter (DS-w) inoculum metabolic profiles to that obtained for the grassland soil summer (GS-s) inoculum (plotted on x-axis).

**Fig. S3.** Microscopic images of bacteria containing PHB inclusions using the lipid Sudan stain and saffranin counterstain. The bacteria strains are (A) *Rhodanobacter sp.* HC50 and (B) *Pseudomonas sp.* HC94. (C) negative control, bacterium with no lipid granules inclusions. The blue dots in the cytoplasma are the lipid-granules, indicated with the arrows. (Nikon Eclipse 80i, oil-immersion objective)

**Fig. S4.** Clear halos appearing on the opaque white plates indicate bacterial phosphorous solubilisation. A phosphate-buffer washed suspension of bacteria, pre-grown in rich medium was inoculated into the holes. All strains were tested in triplicate.

**Fig. S5.** Chemotactic response and flagella stain of *Ralstonia sp.* HC90. (A) Chemotactic response towards aspartic acid and (B) 2,4-DNT. (C) Negative control, lack of chemotaxis of heat-killed cells towards 2,4-DNT. Red arrows indicate the chemotaxis rings. Plates were scanned with a flatbed scanner. (D) Leifson flagella stain of *Ralstonia sp.* HC90, black arrow indicates flagella. Photograph was taken with oil-immersion objective (1500×).

**Fig. S6.** Photographs of the *Arabidopsis thaliana* plants on VAPS-plates (A) control; (B) UHS3 inoculated control; (C) exposed to 2,4-DNT and (D) exposed to 2,4-DNT with UHS3 inoculation, grown at a density of 5 plants per plate. The photograph was taken 9d after transfer of the seedlings when root length measures were taken, root hair pictures and the shoots were collected for reisolation of the inoculated bacteria.

**Fig. S7.** UPGMA dendrogram showing the relationships among the bacteria isolated from *A. thaliana* shoot tissues and the inoculated strains from consortium UHS3.

**Table S1.** Physicochemical characteristics of the 2,4-DNT contaminated soil and adjacent grassland soil. Nitro-aromatic concentrations in the samples were measured with HPLC and are expressed in mg kg ^−1^ dry soil with standard error. EC: Electric Conductivity; 2,4-dinitrotoluene (2,4-DNT); 2,6-dinitrotoluene (2,6-DNT); 1,3-dinitrobenzene (DNB); 1,3,5-trinitrobenzene (TNB); nitrobenzene (NB); 2-amino-4-nitrotoluene (2-A-4NT); 4-amino-2-nitrotoluene (4-A-2NT). ND = not detected (< 0.01 mg l^−1^ ). DS-s: 2,4-DNT contaminated soil summer; DS-w: 2,4-DNT contaminated soil winter; GS-s: grassland soil summer; GS-w: grassland soil winter.

**Table S2.** Fingerprint of the inocula tested with Ecoplates. The absorbance data of the Biolog Ecoplates were blanked to the water containing well and then converted into Boolean ones, considering positive (1) the substrates for which the blanked absorbance data > 0.10 and negative (0) the substrates for which the blanked absorbance data < 0.10. DS-s: 2,4-DNT contaminated soil summer; DS-w: 2,4-DNT contaminated soil winter; GS-s: grassland soil summer; GS-w: grassland soil winter.
