# Supplementary material for: Potential for plant growth promotion by a consortium of stress-tolerant 2,4-dinitrotoluene-degrading bacteria: isolation and characterization of a military soil
Source: Microb Biotechnol. 2014 Jan 28;7(4):294–306. doi: 10.1111/1751-7915.12111 (PMC4241723; doi:10.1111/1751-7915.12111)
Supplement: Supplementary file 1 [file mbt20007-0294-sd1.pdf]

Supplementary information

Supplementary methods

Abiotic stress resistance and plant growth promotion

The ability of the isolated bacteria to cope with abiotic stresses was tested on the 31 strains belonging to the 2,4-DNT tolerant group identified by auxanography. The potential functionality of the isolates to sustain plant growth was assessed *in vitro* by a large screening for PGP-abilities using specific media.

Water stress resistance test. A drop assay was used to test drought resistance of the strains. Drops (10 µl) of cells were spotted onto petri plates and incubated during 14 days at 24°C and allow to dry under ambient humidity. The cells from each drop were suspended separately and the viable count (cfu) was determined. The survival rate was calculated as log (cfu after drying)/log (cfu before drying) x 100 (Alvarez et al., 2004).

N-and C-starvation resistance testing. To measure resistance of the strains under N- and C-limiting conditions, the strains were grown in rich medium for 24 h, two washing steps in $MgSO_4$ were performed and 1 x 10^7 cfu ml^{-1} were inoculated into falcon tubes with 5 ml M8 minimal medium containing 0.2 % glucose and 0.1 g l^{-1} NH_4Cl as sole C and N-source. The strains were grown for 28 d at 24°C. At the end of the cultivation period, the number of cfu ml^{-1} was determined on rich 869 medium. The survival rate was calculated as explained before. The occurrence of lipid granules were screened during the starvation conditions, 1 ml of the culture was centrifuged and the pellet was resuspended in 10 mM $MgSO_4$ and a slight turbid drop of 20 µl was deposited on a glass slide and heat-fixed. Cells were stained with Sudan black B and counterstained with saffranin as described by (López-Cortés et al., 2008).

Heat/cold tolerance. The response of strains to grow at cold and hot temperatures was verified by incubating the strains on 284 minimal medium plates at 4°C and 50°C. Determinations of OD_{600} were performed after 28 days of growth.

In vitro PGP-tests. Indolacetic acid (IAA) was measured according to the protocol described by (Patten and Glick, 2002). Siderophore release was determined by the method of (Schwyn and Neilands, 1987). 1-aminocyclopropane-1-carboxylate (ACC)-deaminase activity was estimated by monitoring the amount of α-ketobutyrate generated by the enzymatic hydrolysis of ACC (Belimov et al., 2005). Organic acid production was determined according to (Cunningham and Kuiack, 1992). Phosphate solubilisation activity was assayed on plates by the method of (Goldstein, 1995). Acetoïn production was measured using the Voges-Proskaur assay (Romick and Fleming, 1998).

Chemotaxis assay. To evaluate chemotaxis of the strains towards 2,4-DNT, we adopted a soft-agar plate assay (Samanta et al., 2000). Briefly, 2,4-Dinitrotoluene (100 mg l^{-1}) was added as sole energy source to selective medium containing 0.5 % Difco Noble agar. Aspartic acid (0.5 g l^{-1}) was used as positive control. The bacteria suspension was poured at the center of the plate. Heat-killed cells were inoculated as negative control. After 24 h of incubation, the plates were observed for formation of growth rings. Subsequently, bacteria were collected with an inoculation loop from the border of the migration rings and stained with the simplified Leifson flagella stain (Clark, 1976).

Root growth promotion under 2,4-DNT stress on VAPS

Wild-type *A. thaliana* seeds were surface sterilized in 0.1 % NaClO for 5 min and washed 4 times with distilled water during 20 min. Seeds were sown on vertical plates with 50 ml of Gamborg's B5/50 medium (1 % Bacto Agar) (Zhang and Forde, 1998). After incubation at 4 °C

for 3 days in the dark, the plates were placed vertically in a plant growth chamber at 22 °C day and 18°C night temperature, with a 12-h/12-h light-dark cycle and photosynthetic active light radiation (PAR) of 180 µmol m^{-2} s^{-1} for 7 days. After 7-days, 5 seedlings were transferred to fresh B5/50 plates amended with 2,4-DNT (0; 5 mg l^{-1}) and whether or not inoculated with the consortia. For the inoculation, overnight cultures of the consortium members were inoculated into fresh 284 medium and growth was monitored. When the culture reached 0.4 OD, cells were centrifuged (4000 rpm), washed twice with 10 mM $MgSO_4$ and 1 ml was combined to reconstitute the consortia. The consortia suspension was brought to OD 1 x 10^7 cfu/ml and 100 µl was spread on the growth plates.

After 9 days, plant primary root length was measured and analyzed using the Optimas 6.1 program (Media Cybernetics). For visualizing root hairs, roots were stained for 1 min in 0.075 % crystal-violet in 70 % ethanol and rinsed thoroughly with distilled water.

At the end of the growth period, reisolation of the bacteria from the endophytic shoot part of the plant was performed. Shoots (50 mg) were surface sterilized for 1 min in 0.1 % Cl-solution followed by 3 washes for 5 min in sterile dH_2O. Surface sterility was confirmed for all samples by plating 100 µl of the last rinsing water onto 869 rich plates and incubating overnight at 30°C. The surface sterilised shoot tissues were crushed in a sterile mortar in 2 ml of sterile 10 mM $MgSO_4$ solution and serial dilutions were plated onto 1/10 rich (869) medium. After 5 days at 30°C, representative colonies per plate were picked for colony box-PCR, performed according to the protocol described by Barac et al., 2004 (Barac et al., 2004). The amplified box-fragments were screened by electrophoresis in 1 % agarose gel and were clustered by Totallab (v2009) using UPGMA method.

Supplementary figures and tables

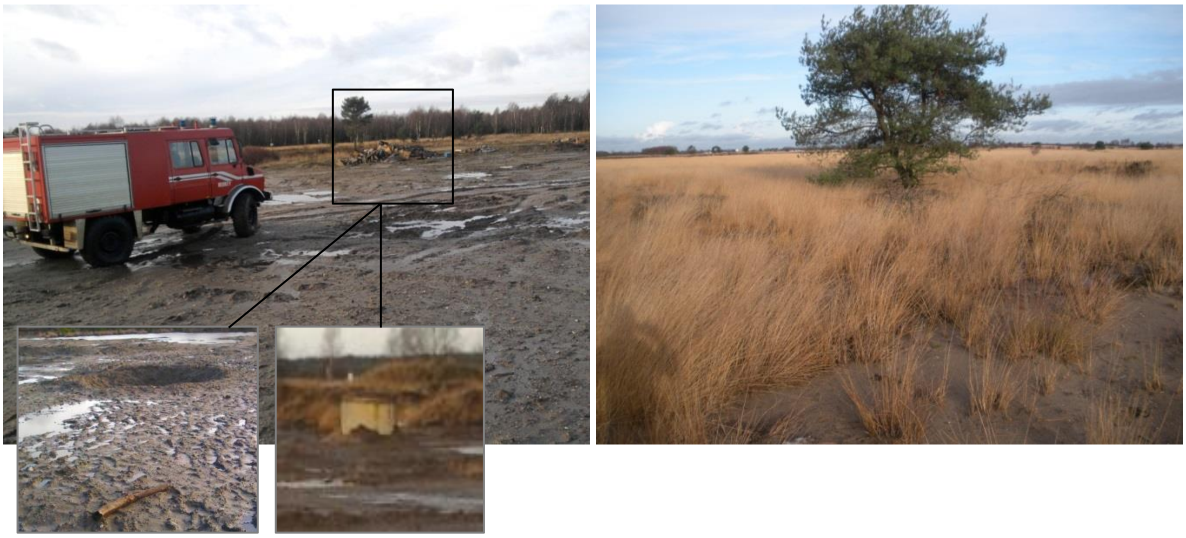

Figure S1: Photographs of the investigated field soils at the military training range in the North-East of Belgium. Left, the bare 2,4-DNT contaminated soil with bomb remnants and detonation container. On the left side, the adjacent grassland soil colonized by *Molinia caerulea* and *Agrostis tenuis*, photograph was taken in November 2009.

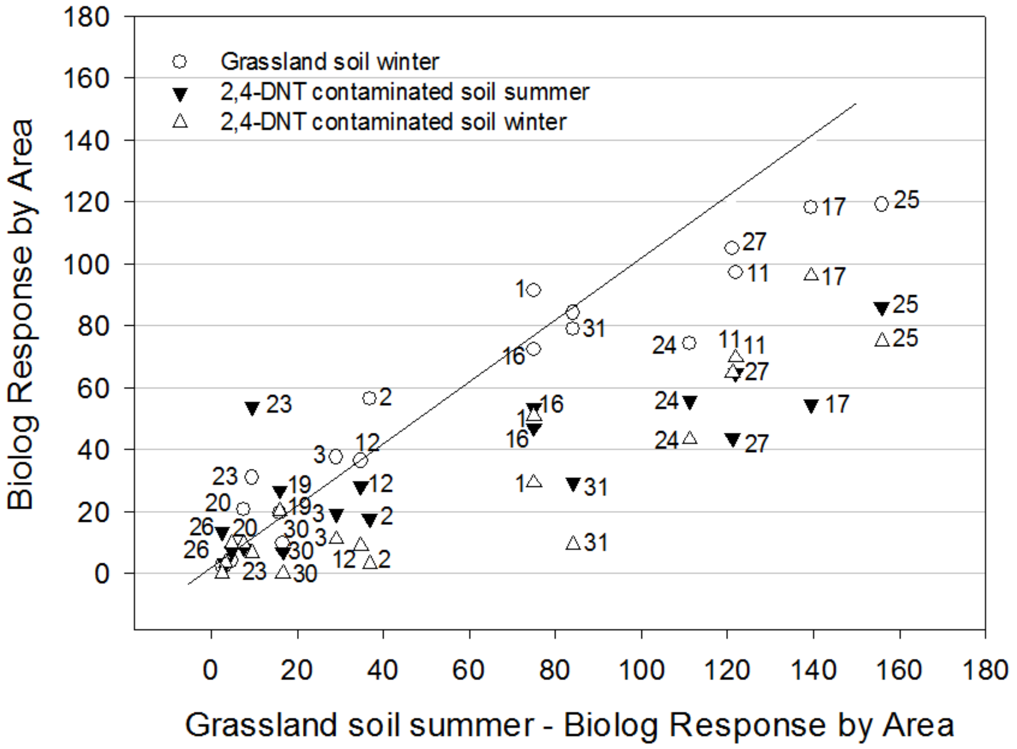

Figure S2: Scatterplot of Biolog EcoPlate responses (average net area under the curve for each of the 31 carbon sources) comparing the grassland soil winter (GS-w), 2,4-DNT contaminated soil summer (DS-s) and winter (DS-w) inoculum metabolic profiles to that obtained for the grassland soil summer (GS-s) inoculum (plotted on x-axis).

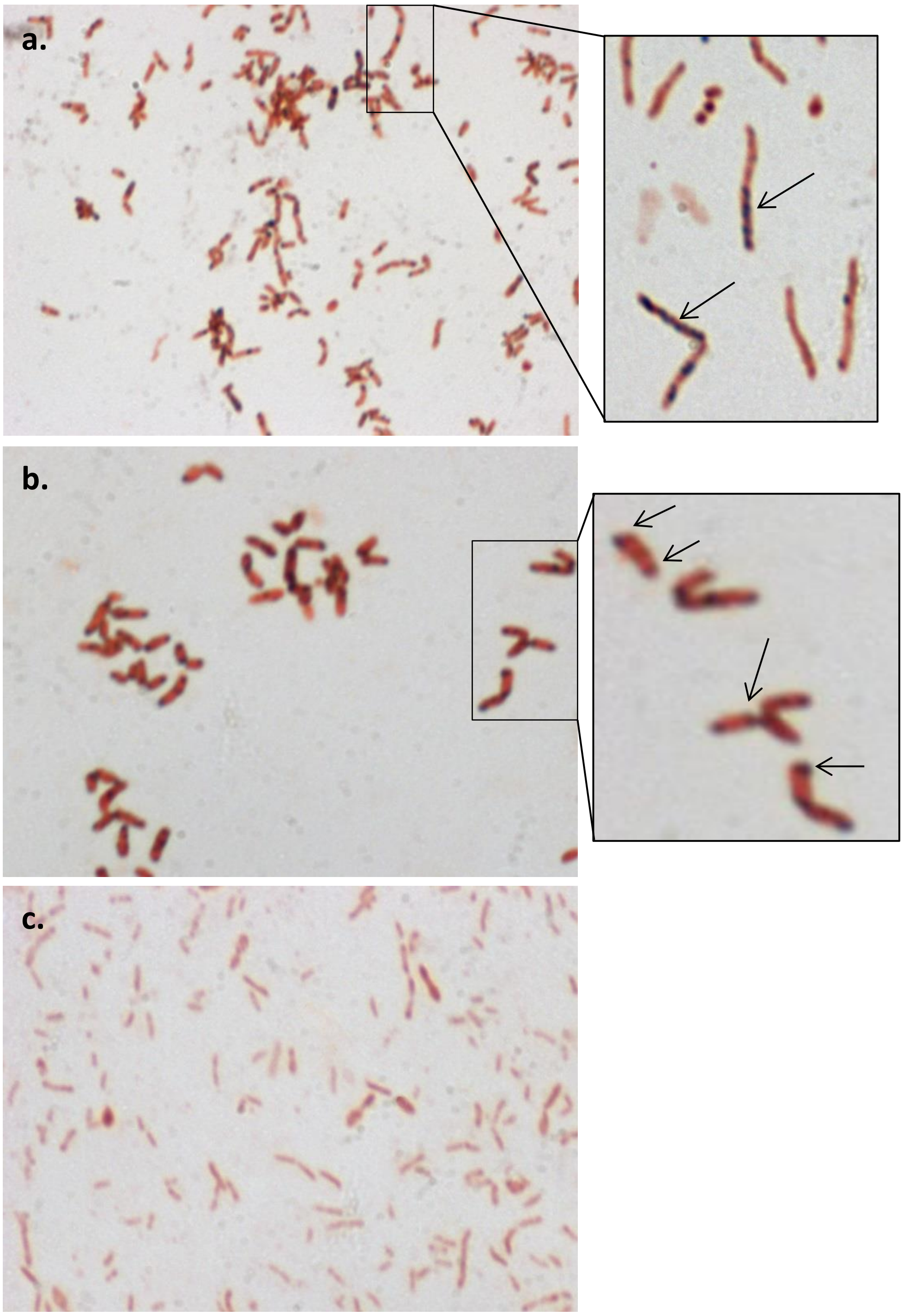

Figure S3: Microscopic images of bacteria containing PHB inclusions using the lipid Sudan stain and saffranin counterstain. The bacteria strains are (**a**) *Rhodanobacter sp.* HC50 and (**b**) *Pseudomonas sp.* HC94. (**c**) negative control, bacterium with no lipid granules inclusions. The blue dots in the cytoplasma are the lipid-granules, indicated with the arrows. (Nikon Eclipse 80i, oil-immersion objective)

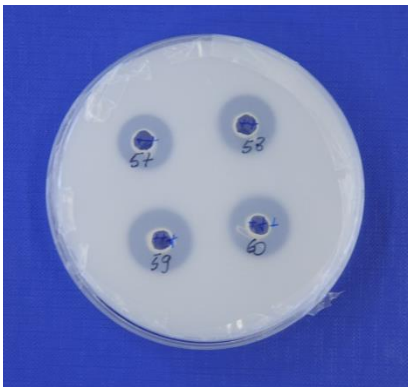

Figure S4: Clear halos appearing on the opaque white plates indicate bacterial phosphorous solubilisation. A phosphate-buffer washed suspension of bacteria, pre-grown in rich medium was inoculated into the holes. All strains were tested in triplicate.

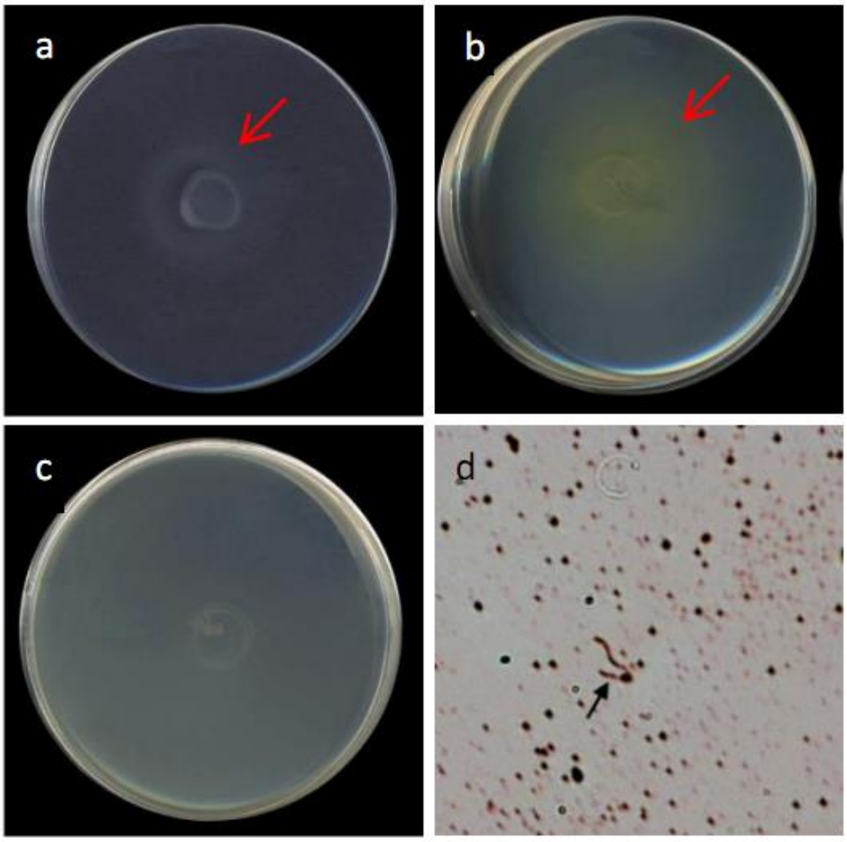

Figure S5: Chemotactic response and flagella stain of *Ralstonia sp.* HC90. (**a**) Chemotactic response towards aspartic acid and (**b**) 2,4-DNT. (**c**) Negative control, lack of chemotaxis of heat-killed cells towards 2,4-DNT. Red arrows indicate the chemotaxis rings. Plates were scanned with a flatbed scanner. (**d**) Leifson flagella stain of *Ralstonia sp.* HC90, black arrow indicates flagella. Photograph was taken with oil-immersion objective (1500 x).

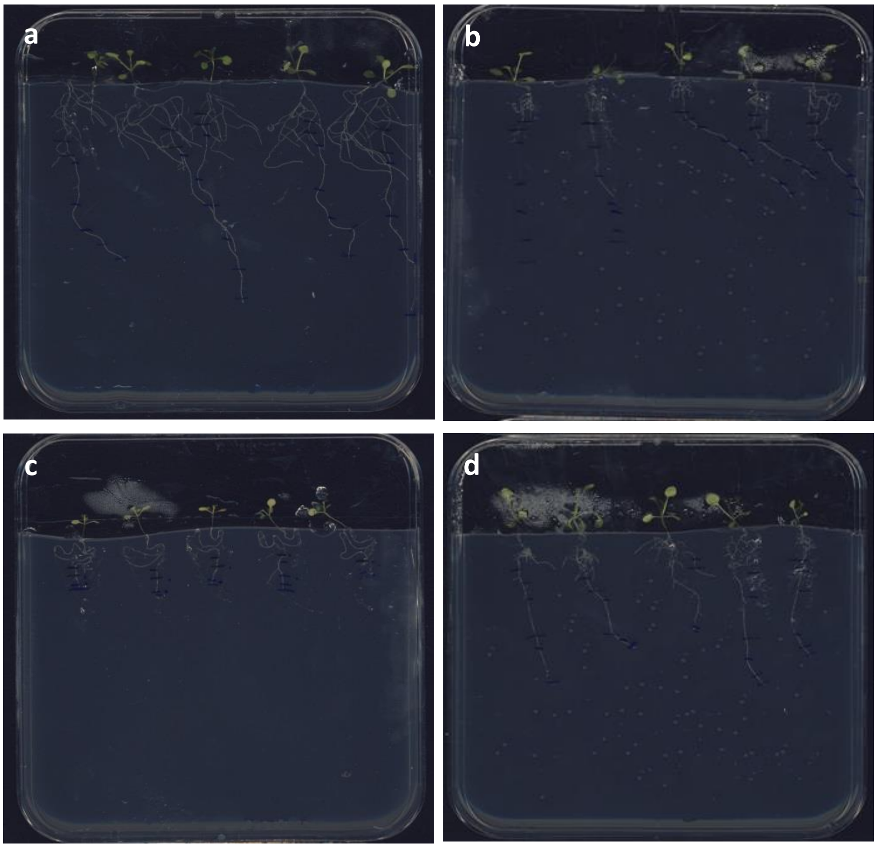

Figure S6: Photographs of the *Arabidopsis thaliana* plants on VAPS-plates (**a**) control; (**b**) UHS3 inoculated control; (**c**) exposed to 2,4-DNT and (**d**) exposed to 2,4-DNT with UHS3 inoculation, grown at a density of 5 plants per plate. The photograph was taken 9d after transfer of the seedlings when root length measures were taken, root hair pictures and the shoots were collected for reisolation of the inoculated bacteria.

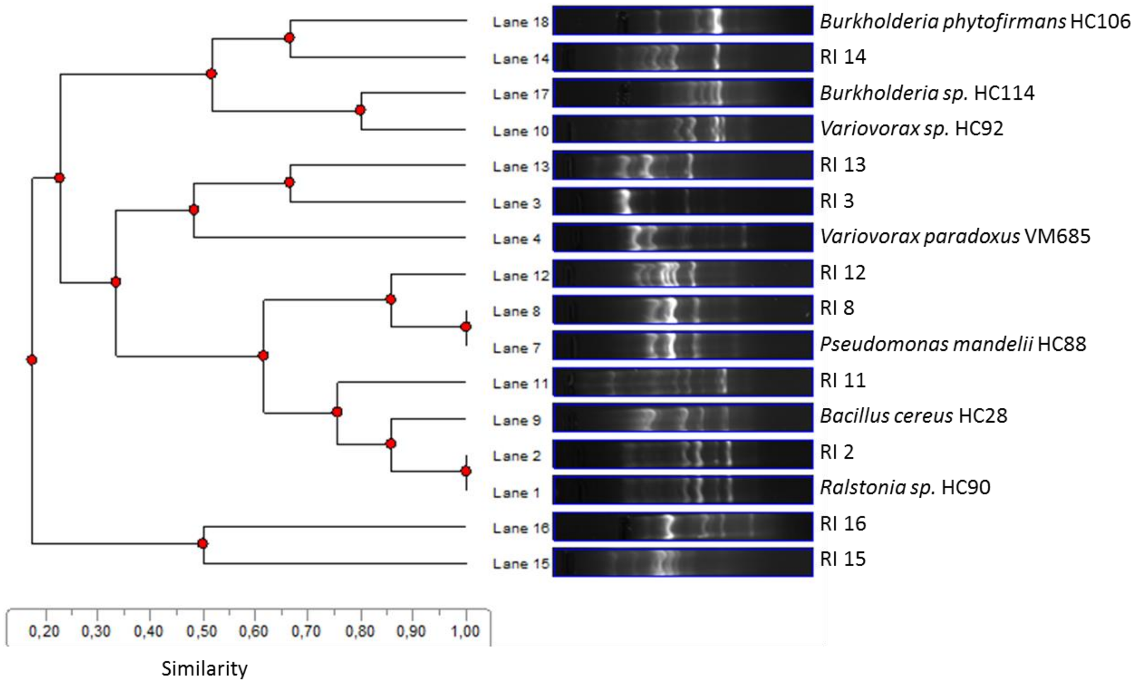

Figure S7: UPGMA dendrogram showing the relationships among the bacteria reisolated (RI) from *A. thaliana* shoot tissues and the inoculated strains from consortium UHS3.

Characteristic	2,4-DNT contaminated soil		adjacent grassland soil	
	**DS-s**	**DS-w**	**GS-s**	**GS-w**
Texture	Sandy soil	Sandy soil	Sandy soil	Sandy soil
pH	6.0	5.6	5.03	5.82
EC (µS cm^{-1})	313	234	240	240
Organic carbon (%)	1.56	1.25	2.13	1.56
2,4-DNT	74.8 ± 9.61	83.4 ± 10.5	1.52 ± 0.56	0.89 ± 1.21
2,6-DNT	3.50 ± 0.61	6.01 ± 1.21	ND	ND
TNB	2.41 ± 0.87	4.21 ± 1.21	ND	0.05 ± 0.01
DNB	1.25 ± 0.12	ND	ND	ND
NB	0.61 ± 0.05	ND	ND	ND
2-A-4NT; 4-A-2NT	19.8 ± 3.44	14.5 ± 1.20	ND	ND

Table S1: Physicochemical characteristics of the 2,4-DNT contaminated soil and adjacent grassland soil. Nitro-aromatic concentrations in the samples were measured with HPLC and are expressed in mg kg^{-1} dry soil with standard error. EC: Electric Conductivity; 2,4-dinitrotoluene (2,4-DNT); 2,6-dinitrotoluene (2,6-DNT); 1,3-dinitrobenzene (DNB); 1,3,5-trinitrobenzene (TNB); nitrobenzene (NB); 2-amino-4-nitrotoluene (2-A-4NT); 4-amino-2-nitrotoluene (4-A-2NT). ND = not detected (<0.01 mg l^{-1}). DS-s: 2,4-DNT contaminated soil summer; DS-w: 2,4-DNT contaminated soil winter; GS-s: grassland soil summer; GS-w: grassland soil winter.

	Eco-substrates	Microbial inocula			
		DS-s	DS-w	GS-s	GS-w
1	Pyruvic acid methyl ester	1	1	1	1
2	Tween 40	1	0	1	1
3	Tween 80	1	1	1	1
4	α-Cyclodextrin	1	1	1	1
5	Glycogen	1	0	1	1
6	D-Cellobiose	0	0	0	0
7	α-D-Lactose	0	0	0	0
8	β-Methyl-D-glucoside	0	0	0	1
9	D-Xylose	0	0	1	1
10	i-Erythritol	1	1	1	1
11	D-Mannitol	1	1	1	1
12	N-acetyl-D-glucosamine	1	1	1	1
13	D-Glucosaminic acid	1	1	1	1
14	Glucose-1-phosphate	0	0	0	1
15	D,L-α-Glycerol phosphate	1	0	1	1
16	D-Galactonic acid γ-lactone	1	1	1	1
17	D-Galacturonic acid	1	1	1	1
18	2-Hydroxybenzoic acid	0	0	0	0
19	4-Hydroxybenzoic acid	1	1	1	1
20	γ-Hydroxybutyric acid	1	1	1	1
21	Itaconic acid	1	1	1	1
22	α-Ketobutyric acid	0	0	0	0
23	D-Malic acid	1	0	1	1
24	L-Arginine	1	1	1	1
25	L-Asparagine	1	1	1	1
26	L-Phenylalanine	1	1	1	1
27	L-Serine	1	1	1	1
28	L-Threonine	1	1	1	1
29	Glycyl-L-glutamic acid	1	1	1	1
30	Phenylethylamine	1	0	1	1
31	Putrescine	1	1	1	1

Table S2: Fingerprint of the inocula tested with Ecoplates. The absorbance data of the Biolog Ecoplates were blanked to the water containing well and then converted into Boolean ones, considering positive (1) the substrates for which the blanked absorbance data > 0.10 and negative (0) the substrates for which the blanked absorbance data < 0.10. DS-s: 2,4-DNT contaminated soil summer; DS-w: 2,4-DNT contaminated soil winter; GS-s: grassland soil summer; GS-w: grassland soil winter.

References

Alvarez, H.M., Silva, R.A., Cesari, A.C., Zamit, A.L., Peressutti, S.R., Reichelt, R. et al. (2004) Physiological and morphological responses of the soil bacterium Rhodococcus opacus strain PD630 to water stress. *FEMS Microbiol Ecol* **50**: 75-86.

Barac, T., Taghavi, S., Borremans, B., Provoost, A., Oeyen, L., Colpaert, J.V. et al. (2004) Engineered endophytic bacteria improve phytoremediation of water-soluble, volatile, organic pollutants. *Nature Biotechnology* **22**: 583-588.

Belimov, A.A., Hontzeas, N., Safronova, V.I., Demchinskaya, S.V., Piluzza, G., Bullitta, S., and Glick, B.R. (2005) Cadmium-tolerant plant growth-promoting bacteria associated with the roots of Indian mustard (*Brassica juncea L. Czern*.). *Soil Biology & Biochemistry* **37**: 241-250.

Clark, W.A. (1976) Simplified Leifson flagella stain. *Journal of Clinical Microbiology* **3**: 632-634.

Cunningham, J.E., and Kuiack, C. (1992) Production of citric and oxalic acids and solubilization of calcium phosphate by Penicillium bilaii. *Appl Environ Microbiol* **58**: 1451-1458.

Goldstein, A.H. (1995) Recent Progress in Understanding the Molecular Genetics and Biochemistry of Calcium Phosphate Solubilization by Gram Negative Bacteria. *Biological Agriculture & Horticulture* **12**: 185-193.

López-Cortés, A., Lanz-Landázuri, A., and García-Maldonado, J. (2008) Screening and Isolation of PHB-Producing Bacteria in a Polluted Marine Microbial Mat. *Microbial Ecology* **56**: 112-120.

Patten, C.L., and Glick, B.R. (2002) Role of Pseudomonas putida Indoleacetic Acid in Development of the Host Plant Root System. *Applied and Environmental Microbiology* **68**: 3795-3801.

Romick, and Fleming (1998) Acetoin production as an indicator of growth and metabolic inhibition of Listeria monocytogenes. *Journal of Applied Microbiology* **84**: 18-24.

Samanta, S.K., Bhushan, B., Chauhan, A., and Jain, R.K. (2000) Chemotaxis of a Ralstonia sp SJ98 toward different nitroaromatic compounds and their degradation. *Biochemical and Biophysical Research Communications* **269**: 117-123.

Schwyn, B., and Neilands, J.B. (1987) Universal chemical assay for the detection and determination of siderophores. *Anal Biochem* **160**: 47-56.

Zhang, H.M., and Forde, B.G. (1998) An Arabidopsis MADS box gene that controls nutrient-induced changes in root architecture. *Science* **279**: 407-409.
